# Presentation Patterns, Diagnostic Markers, Management Strategies, and Outcomes of IgD Multiple Myeloma: A Systematic Review of Literature

**DOI:** 10.7759/cureus.4011

**Published:** 2019-02-04

**Authors:** Insija I Selene, Jemin Aby Jose, Muhammad J Khalil, Muhammad Salman Faisal, Mustafa N Malik

**Affiliations:** 1 Internal Medicine, The University of Arizona, Tucson, USA; 2 Internal Medicine, The University of Arizona, Tucson , USA; 3 Internal Medicine, Allegheny Health Network, Pittsburgh, USA

**Keywords:** igd multiple myeloma, bortezomib, overall survival, n-glycan biomarker

## Abstract

Immunoglobulin (Ig) D multiple myeloma (MM) is a rare subtype of MM comprising 2% of all the cases. Malignant plasma cell invasion leads to signs and symptoms similar to other subtypes of MM. The synthesis rate of IgD is lower in IgD MM patients, making it very difficult to diagnose compared to other subtypes. As there is no available diagnostic test with 100% accuracy, the diagnosis of IgD MM is based on multiple factors. Recent advances in the treatment have resulted in a better overall survival for IgD MM patients. The aim of this systematic review was to summarize the data on presentation patterns, diagnosis modalities, management strategies, and outcomes in patients with IgD MM.

## Introduction and background

Multiple myeloma (MM) is the malignant clonal proliferation of plasma B cells in the bone marrow. These malignant plasma cells invade multiple organs producing various manifestations including bone pain, renal failure, hypercalcemia, fractures, anemia and hyperviscosity symptoms [1-2]. Criteria for the diagnosis of MM include ≥10% abnormal plasma B cells in bone marrow, monoclonal proteins (M-proteins) in serum and urine electrophoresis, and clinical features of MM. About 10% of all the hematological cancers and 1% of all cancers are represented by MM [3]. MM can be of various types involving different isotypes of immunoglobulin (Ig) heavy chains and immunoglobulin light chains [4]. IgG, IgA, and light chain myelomas are the most prevalent ones comprising 54%, 21%, and 16% of all the myelomas, respectively [5]. IgD MM being a rare isotype comprises less than 2% of all MM cases [6]. Malignant plasma cell invasion associated with IgD MM, like other subtypes of MM, leads to osteolytic lesions, extramedullary involvement, amyloidosis, renal failure, hypercalcemia, and Bence Jones proteinuria (BJP). IgD has a half-life of 2.8 days and accounts for 0.25% of the total serum immunoglobulins. The synthesis rate of IgD is at least 10 times lower than that of IgA, IgM, and IgG. The patients with IgD myeloma have a poor outcome when compared with other subtypes, with a median survival between 13 and 21 months [7]. The recent advances in the treatment of MM have improved the outcomes in IgD MM patients, though it is considered to have an aggressive course [6]. The aim of this systematic review was to summarize the data on presentation patterns, diagnostic modalities, management strategies, and the respective outcomes in IgD MM patients.

## Review

Material and methods

**Figure 1:**
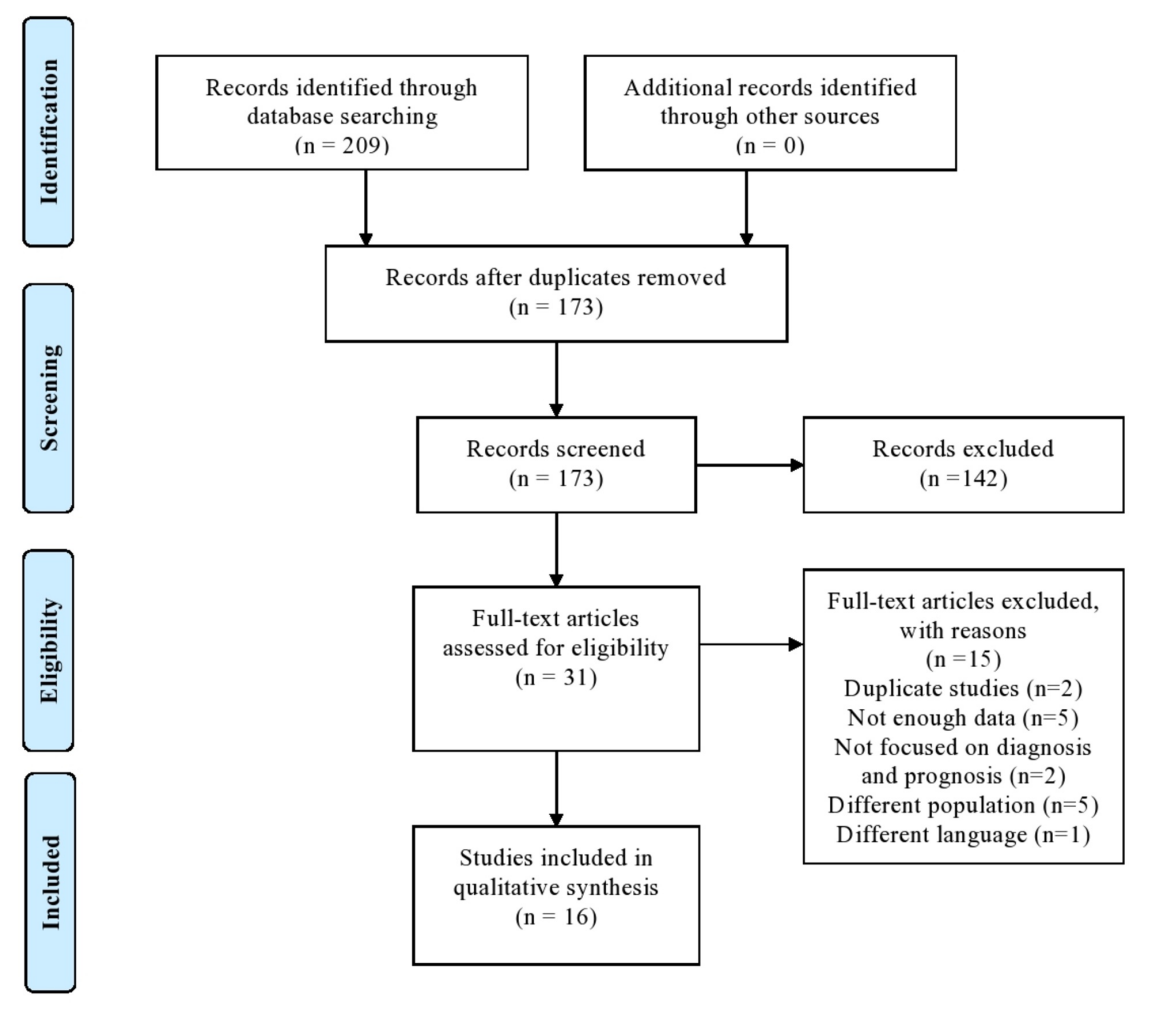
Data identification, screening, eligibility testing, and inclusion according to PRISMA guidelines PRISMA: preferred reporting items for systematic reviews and meta-analyses

A systemic review was performed according to the Preferred Reporting Items for Systematic Reviews and Meta-Analyses (PRISMA) statement for reporting systemic reviews (Figure 1). After formulating the study question, controlled vocabulary search terms (Medical subject headings [MeSH] and Embase subject headings [Emtree]) along with keywords were used to search the studies that examined IgD MM in following five databases: PubMed/Medline, Elsevier/Embase, Web of Science, Wiley/Cochrane library, and ClinicalTrials.gov. The search was conducted for studies after December 2013 with language limitation to English-only studies. The literature search was last updated on May 7, 2018. Reference list along with the respective citations of selected articles was screened for any additional relevant studies.

Two independent reviewers initially screened all retrieved titles and abstracts for relevance. The same protocol was used to screen the selected articles for full texts to check their relevance. Disagreements were resolved by consensus. We finalized 16 studies from a total of 209 articles that were found on initial search.

Inclusion criteria for our review were as follows: (1) Patients with IgD MM, disease patterns, and presentations; (2) case series and case reports with any therapeutic intervention of IgD MM; and (3) studies reporting the efficacy of treatment in terms of median overall survival (mOS), overall survival (OS), complete remission (CR), or very good partial response (VGPR). Exclusion criteria are (1) articles in which other isotypes of MM were studied; (2) opinions, reviews including systemic reviews, and meta-analyses; and (3) articles not in English.

Two independent authors extracted the data, which were subsequently examined by other authors to settle discrepancies. The following variables were analyzed: author, year, study design, number of subjects, laboratory findings, treatment received after the diagnosis of IgD MM, and survival outcomes mainly overall survival or response. This work was presented as a poster in the American Society of Hematology Annual Meeting, 2018 (Poster: Selene II, Jose JA, Khalil MJ, Tariq MJ, Durer S, Durer C, et al. Presentation Pattern, Diagnostic Markers, Management and Outcome of IgD Multiple Myeloma: A Systematic Review. American Society of Hematology Annual Meeting; December 2, 2018).

Results

One hundred sixty-six (166) patients with IgD MM were included. One hundred thirty-six (81.9%) patients had lambda (λ) as their light chain subtype, and 30 (18.1%) patients had kappa (κ) as their light chain subtype. The median age of the patients was 54.5-65 years. There were a total of 104 (62.7%) males and 62 (37.3%) females, with a male:female ratio of 1.68:1.

The reported initial manifestations were Bence Jones proteinuria in 64.5%, renal dysfunction in 63%, bone pain in 55.9%, generalized weakness, and fatigue in 34.2%, and extramedullary involvement in 28.3% patients. During the progression of the disease, 69.8% patients had anemia, 47.1% patients had infections, 38.8% patients had renal failure, 13% of the patients had pleural effusion, and 3.5% patients had amyloidosis. Poor renal function assessed as low estimated glomerular filtration rate (eGFR <60 ml/min/1.73 m^2^) was seen in 54.3% of patients and by elevated serum creatinine levels (Cr >2 mg/dl) in 46.1% patients. New osteolytic lesions were seen in 66.2% of patients by imaging studies out of 71 evaluable patients. The patient characteristics and major lab parameters associated with IgD MM are mentioned in Table 1 and Table 2.

**Table 1:** Patient characteristics and disease manifestations IgD: immunoglobulin D; ISS: international staging system; N: number of patients; S/S: sign and symptoms

IgD multiple myeloma	N (%)
Total patients	166
Lambda subtype	136 (81.9%)
Kappa subtype	30 (18.1%)
Gender	
Male (M)	104 (62.7%)
Female (F)	62 (37.1)
M:F ratio	1.68:1
Median age	54.5-65 years
ISS staging	
Stage I	21 (14.3%)
Stage II	36 (24.5%)
Stage III	90 (61.2%)
Abnormal cytogenetics	41 (73.2%)
S/S at initial presentation	
Bence jones proteinuria	64.5%
Renal dysfunction	63%
Bone pain	55.9%
Weakness and fatigue	34.2%
Extramedullary involvement	28.3%
Complications	
Anemia	69.8%
Infections	47.1%
Renal failure	38.8%
Pleural effusion	13%
Amyloidosis	3.5%

**Table 2:** Deranged lab parameters in IgD MM with their percentages in our review IgD: immunoglobulin D, MM: multiple myeloma, µg: microgram, dl: deciliter, ml: milliliter, mg: milligram, U/L: units per liter

Lab parameters	Percentage of patients
β2 Microglobulin >5 µg/ml	62.5%
Hypocalcemia	50%
Thrombocytopenia	41%
Serum ldh >300 u/l	30.3%
Serum albumin <3.5 mg/dl	27.6%
Hypercalcemia	9.5%
Pancytopenia	5.8%

International Staging System (ISS) staging was done in 147 (88.6%) patients at their presentation, out of which, 61.2% of patients had stage III disease, 24.5% of patients had stage II disease, and 14.3% of patients had stage I disease. Cytogenetic karyotype analysis in 56 patients using fluorescence in-situ hybridization (FISH) identified abnormal cytogenetics in 41 (73.2%) patients who were classified as high risk (85.7%) and standard risk (14.3%).

Serum protein electrophoresis (SPEP) showed a positive monoclonal (M) spike in 84% of the patients. Disturbed free light chain ratio (Sflcr) was seen in 83% patients. Quantitative serum IgD levels were elevated only in 28% of the patients. Bone marrow (BM) plasmacytosis of >40% abnormal plasma cells was detected in 95.6% patients. N-glycans are newly discovered biomarkers used for detecting abnormal protein glycosylation. NG1(6)A2F and NG1(3)A2F are the two most significant N-glycan markers with the sensitivity of 95% & 95.2%, respectively, and specificity of 95% & 78.6%, respectively [8].

Details on treatment were available for 149 patients and overall response rate (ORR) was seen in 125 (83.9%) patients, while 24 (15.4%) patients had progressive or stable disease. The median overall survival was nine to 62 months. Novel agents (NA = bortezomib, thalidomide, and lenalidomide) were given to 111 (74.4%) patients, resulting in the mOS of 23 months (range = 15-38.6). Conventional agents (CA = melphalan, vinblastine, vincristine, epirubicin, and ifosfamide) were given to 38 (25.5%) patients, resulting in the mOS of 14 months (range = 12.5-17). Data showed that bortezomib-based regimens showed a higher overall response rate (ORR = 94%, CR = 52%) when compared to non-bortezomib-based regimens (ORR = 77.8%, CR = 27.7%). Autologous stem cell transplantation was given to 37 patients along with chemotherapy (NA = 26 patients, CA = 11 patients) which showed good response rate (ORR >90%, CR >60%; Table 3).

**Table 3:** Response rate of drug regimens CR: complete response; mOS: median overall survival; mo: months; mPFS: median progression-free survival; n: number of patients; ORR: overall response rate; PR: partial response; VGPR: very good partial response

Drugs	ORR	CR	VGPR	PR	mPFS (months)	mOS (months)
Without stem cell transplant (n = 149)						
Novel agents (n = 111)	91%	44.8%	20.5%	25.6%	16	23
Bortezomib-based (n = 80)	94%	52%	22%	20%	18	23
Non-bortezomib based (n = 31)	77.8%	27.7%	16.7%	33.3%	12	21
Conventional agents (n = 38)	91.6%	25%	8.3%	58.3%	N.S.	14
With stem cell transplant (n = 37)						
Novel agents (n = 26)	95.7%	60.9%	30.4%	4.3%	8.3	31
Conventional agents (n = 11)	91.7%	66.7%	16.7%	8.3%	7.4	12.5

Discussion

IgD-secreting plasma cells are the product of somatic hypermutation of the IgD region of germinal center B cells [5]. The various subtypes based on serum free light chain analysis are 70% to 90% lambda (λ) and 3% to 4% kappa (κ), showing λ predominance over κ light chains in contrast to other subtypes [9-11]. This predominance of λ light chains can be due to preferential rearrangements of genes of heavy chains λ with light chains λ [4]. There was a difference in mOS and progression-free survival (PFS), with increased survival (4-11mo) in λ subtype compared to κ subtype, but the data were not statistically significant (*p *> 0.05) [6]. IgD MM has been found involving relatively younger patient population, predominantly males, with an average age of 59 years (54-65 years) [4,7,12]. Fatigue, weakness, pallor, and bone pain are the most common initial manifestations. However, patients usually present in advanced ISS stage with clinical features such as renal dysfunction, Bence Jones proteinuria (BJP), osteolytic lesions, extramedullary involvement, amyloidosis, and renal failure [4-5,13]. Zagouri et al. (2014) reported that renal dysfunction and BJP are higher for IgD subtype compared to other subtypes of MM (*p *< 0.001) [7]. BJP is a frequent manifestation in different studies of IgD MM (71%) but is present only in 35% of cases in IgG MM and 20% of cases in IgA MM [4]. Extra-medullary involvement is seen in 19% to 63% of patients with usual sites being the chest wall, respiratory tract, gastrointestinal tract (GI) tract, skin, lymph nodes, paraspinal areas, and rarely isolated testicular involvement [5].

Renal failure was present in 20% to 40% of patients at the time of diagnosis [14]. The mechanisms of renal injury can be either due to light chain cast nephropathy or through the direct toxicity caused by intracellular crystals [1,15]. AL amyloidosis, mostly affecting heart (45%), is a common complication in IgD MM (19%) compared to IgG (5%), IgA (2%), and light chain MM (LCMM) (13%) as revealed by Mayo Clinic series [4]. There was no significant difference (*p *> 0.05) between the survival of patients with IgD MM with and without associated AL amyloidosis.

High-risk chromosomal abnormalities in patients with IgD MM have been reported to range from 33% to 53% in various studies. The high-risk anomalies include t(4:14), t(14: 16), 1q21 amplification, del 17, p53, IgH translocation. The mOS was 14.5 months (range: 5-68) in the group with 1q21 amplification and 18 months (range: 8-36) in the group without 1q21 amplification. There were no significant differences in OS and PFS between the two groups (*p* = 0.730 and *p *= 0.185, respectively) [12].

Diagnosis

The diagnostic panel of IgD MM confirms the presence of M protein in MM and determines its isotype. The concentration of monoclonal peaks on electrophoresis is lower in IgD MM (median = 9.42 g/L) compared to those found in IgG (median: 35 g/L) and IgA (median: 32 g/L) due to the small amount of physiological IgD [4]. Quantitative serum IgD can be normal or lower than normal range in some cases [6]. The lower serum concentration of IgD i.e. 0-10 mg/dL compared to 1020-1460 mg/dL in IgG and 210-350 mg/dL in IgA is responsible for the false-negative results on electrophoresis [8]. This can lead to misdiagnosis of IgD MM as a non-secretory MM (NSMM). Rarely, a subtype of NSMM can class switch to IgD MM [16].

N-glycans are newly discovered biomarkers used for detecting abnormal protein glycosylation. NG1(6)A2F and NG1(3)A2F are the two most significant N-glycan markers with the sensitivity of 95% & 95.2%, respectively, and specificity of 95% & 78.6%, respectively. A study conducted by Chen et al. (2017) identified the role of N-glycan biomarkers in the diagnosis and prognosis of IgD MM using DNA sequencer-assisted fluorophore-assisted carbohydrate electrophoresis (DSA-FACE) technique by sensitive detection of serum N-glycosylation changes and abnormalities (Table 4) [8]. There were 12 N-glycan peaks in IgD myeloma with higher peaks contributed by NA2FB, NA3, NA3FB, NA3F2, NA4, and NA4FB. The clinically significant ones are NA2FB, NG1(6)A2F, and NG1(3)A2F with prognostic and diagnostic value. NA2FB above the median level is associated with poor prognosis. False-negative SPEP and IFE results could be avoided using these supplemental markers.

**Table 4:** Role of N-glycan in the diagnosis and prognosis of IgD multiple myeloma Acc: accuracy; IPE: immune electrophoresis; OS: overall survival; PFS: progression-free survival; Sn: sensitivity; Sp: specificity; SPEP: serum protein electrophoresis

Number of N-glycan peaks	12	
Characteristics		
Significantly higher N-Glycan peaks	NA2, NA3, NA3FB, NA3F2, NA4, NA4FB	
Difference in peak level in κ and λ subjects	Nil	
Staging		
ISS staging	Inversely proportional to NG1 (3)A2F peak	p = 0.011
DSS staging	Directly proportional to NA3F2 peak	p = 0.036
Lab parameters		
Positive SPEP and IP	Higher NA2FB	p = 0.001
Negative SPEP and IP	Lower NA2FB	P = 0.036
Diagnostic tool		
NG1(6)A2F	Sn 95% Sp 95.2% Acc 95.1%	
NG1(3)A2F	Sn 95% Sp 78.6% Acc 86.8%	
P		
PFS	Inversely proportional to NA2FB level	p = 0.008
OS	Inversely proportional to NA2FB level	p ≥ 0.05

Treatment

Treatment of IgD MM is similar to other subtypes of MM. Novel agents have shown an increase in the overall survival [6,12,17-18]. However, Liu et al. showed a decrease in overall survival by two months in the patient treated with novel agents compared to non-novel agents [19]. Progression-free survival (PFS) was reported in only a few articles with better PFS in patients treated with novel agents [17]. Bortezomib-based regimens were the most frequently used (86%) novel therapy with excellent efficacy. Studies comparing bortezomib-based regimens with non-bortezomib-based regimens showed higher (1.5-2 months) overall survival and PFS with bortezomib-based regimens [12]. The use of high-dose consolidation chemotherapy with novel agents has shown a better overall survival in patients with IgD MM [12,20]. Kang et al. showed poor response rate after stem cell transplantation (SCT) in the novel agent group but no change in response in the non-novel agent group, while the study conducted by Wang et al. showed an increase in the overall response rate after SCT [12,17].

Prognosis

Monitoring of the IgD MM can be performed with serum heavy IgD quantification along with serum free light chain (FLC) assay and immunofixation electrophoresis (IFE) [9]. Various disease factors and their relationship with the prognosis of IgD MM are mentioned in Table 5. The literature review revealed that patients with IgD MM have a shorter overall survival (nine months) compared to patients with IgG (49 months), IgA (40 months), and light chain MM (35 months) [4,21]. In a multivariate model adjusted for differences in prognostic features, IgD myeloma was not associated with a different prognosis compared to other subtypes of MM (HR: 0.965, 95% CI 0.56-1.45, *P* = 0.887) [4].

**Table 5:** Evaluation of various prognostic factors in IgD multiple myeloma amp: amplification; FLC: free light chain; LCR: light chain ratio; mOS: median overall survival; N: normal; NS: not specified; OS: overall survival; PFS: progression-free survival.

Biomarker	Comparison (months)	P-value	Author, year	Number of patients
Statistically significant (P < 0.05)				
Serum-free LCR – N/abnormal	mOS: 12 /3	0.03	Djidjik et al., 2015 [4]	17
Serum IgD – N/high	PFS: NS	0.022	He J et al., 2016 [21]	29
Serum IgD quantification + Serum FLC levels – N/abnormal	PFS: 7.8/43.9	0.03	He J et al., 2016 [21]	29
Bone marrow plasmacytosis – higher percentage	OS: NS	0.03	Wang et al., 2016 [12]	68
N glycan peak – Below/Above median level	PFS: 27/10.9	<0.008	Chen J et al., 2017 [8]	20
Statistically non-significant (P > 0.05)				
Cytogenetics – No 1q21 amp/1q 21 amp	PFS: 18 /13	0.185	Wang et al., 2016 [12]	68

## Conclusions

IgD myeloma is a rare subtype of MM. The clinical presentation of IgD MM is similar to the other myeloma subtypes, but the diagnosis may be delayed due to the lower prevalence and smaller volume of IgD monoclonal proteins in the serum. The treatment of IgD MM is the same as the other subtypes of MM. Bortezomib-based regimens along with stem cell transplantation are found to be the most effective treatment. More studies involving larger populations are needed to further explore the disease and improve patient outcomes.

## References

[1] Gao L, Li Q, Kang J, Li C, Zhou J (2015). Non-secreting multiple myeloma switches to IgD of lamda type: a case report and review of literature. Int J Clin Exp Med.

[2] Kyle RA, Rajkumar SV (2009). Criteria for diagnosis, staging, risk stratification and response assessment of multiple myeloma. Leukemia.

[3] Rebecca S, Deepa N, Ahmedin J (2013). Cancer statistics. CA Cancer J Clin.

[4] Djidjik R, Lounici Y, Chergeulaine K (2015). IgD multiple myeloma: clinical, biological features and prognostic value of the serum free light chain assay. Pathol Biol (Paris).

[5] Sharma A, Binazir T, Sintow A, Lee CC, Shaharyar S, Tache J (2017). An extremely rare manifestation of multiple myeloma: an immunoglobulin D secreting testicular plasmacytoma. Cureus.

[6] Ongoren S, Erdogan I, Salihoglu A (2015). IgD multiple myeloma, descriptive report of eight cases, single centre experience. Clin Lymphoma Myeloma Leuk.

[7] Zagouri F, Kastritis E, Symeonidis AS (2014). Immunoglobulin D myeloma: clinical features and outcome in the era of novel agents. Eur J Haematol.

[8] Chen J, Fang M, Chen X (2017). N-glycosylation of serum proteins for the assessment of patients with IgD multiple myeloma. BMC Cancer.

[9] Yang W, Zhang X-J, Wang H-C, Yang G-Y, Xu X-F (2018). Case report IgD-λ multiple myeloma accompanying with elevated AFP level: a case report and literature review. Int J Clin Exp Med.

[10] De Santis E, Masi S, Cordone I (2016). Follow-up of IgD-kappa multiple myeloma by monitoring free light chains and total heavy chain IgD: A case report. Oncol Lett.

[11] Laribi K, Lemaire P (2017). IgD kappa multiple myeloma and myelodysplastic syndrome. Blood.

[12] Wang GR, Sun WJ, Chen WM (2016). Immunoglobulin D multiple myeloma: disease profile, therapeutic response, and survival. Acta Haematol.

[13] Modi J, Kamal J, Eter A, El-Sayegh S, El-Charabaty E (2015). Immunoglobulin D multiple myeloma with rapidly progressing renal failure. J Clin Med Res.

[14] Rabrenovic V, Mijuskovic Z, Marjanovic S (2015). Kidney failure as an unusual initial presentation of biclonal gammopathy (IgD multiple myeloma associated with light chain disease)--a case report. Vojnosanit Pregl.

[15] Lescoat A, Rioux-Leclercq N, Vigneau C (2015). Demonstration of the cause of acute renal failure in a case of IgD multiple myeloma. Br J Haematol.

[16] Robier C, Piribauer M, Beham-Schmid C, Aubell K, Neubauer M (2017). IgD-lambda myeloma with extensive free light-chain excretion: a diagnostic pitfall in the identification of monoclonal gammopathies. Clin Chem Lab Med.

[17] Kang J, Hong JY, Yoon DH (2018). Efficacy and survival outcome associated with the use of novel agents and autologous stem cell transplantation in cases of immunoglobulin D multiple myeloma in Korea. Acta haematol.

[18] Gale RP (2018). Therapy for immunoglobulin D plasma cell myeloma. Acta haematol.

[19] Liu Y, Ke XY, Wang J (2014). Clinical characteristics and therapeutic efficacy of immunoglobin D multiple myeloma. J Exp Hematol.

[20] Husnain M, Kurtin S, Barkett N, Riaz IB, Agarwal A (2016). Refractory IgD multiple myeloma treated with daratumumab: a case report and literature review. Case Rep Oncol Med.

[21] He J, Zhang H, Jiang H, Zeng T, Chang H, Hou J (2016). The significance of serum IgD quantitation for evaluation of clinical efficacy in IgD multiple myeloma. Chin J Hematol.

